# Association between body fat distribution and B-lymphocyte subsets in peripheral blood

**DOI:** 10.1186/s12979-023-00372-6

**Published:** 2023-09-13

**Authors:** Pia Prechtl, Timo Schmitz, Nicole Pochert, Claudia Traidl-Hoffmann, Jakob Linseisen, Christa Meisinger, Dennis Freuer

**Affiliations:** 1 Epidemiology, Medical Faculty, University of Augsburg, Augsburg, Germany; 2 Institute for Medical Information Processing, Biometry and Epidemiology – IBE, LMU, Munich, Munich, Germany; 3 Institute of Environmental Medicine, Helmholtz Munich, Munich, Germany; 4 Department of Obstetrics and Gynecology, University Hospital of Augsburg, Augsburg, Germany; 5 Environmental Medicine, Faculty of Medicine, University of Augsburg, Augsburg, Germany

**Keywords:** Visceral fat, Adaptive immunity, B cells, B-lymphocytes, Immune system, Obesity

## Abstract

**Background:**

Obesity is associated with chronic low-grade inflammation, which is underpinned by the presence of elevated levels of circulating proinflammatory cytokines in obese individuals. Due to the close relationship between adipose tissue and the immune system, it can be speculated that the accumulation of fat may influence the frequency and phenotype of lymphocyte populations. The aim of our study was to investigate whether body fat distribution is associated with B lymphocyte composition in peripheral blood. We examined the association between visceral (VAT) and total body fat (TBF) and the frequencies of B-cell subsets in 238 subjects over a period of up to one year using random intercept models. B lymphocyte subsets were determined by fluorescence-based flow cytometry.

**Results:**

Inverse associations were found between body fat measurements and plasma blasts, memory B cells, and IgM^**−**^IgD^**−**^ cells. VAT, but not TBF, was positively associated with naive CD19 cells. In our analyses, both VAT and TBF showed positive associations with IgD only B cells.

**Conclusions:**

In conclusion, body fat accumulation seems to be associated with a lower proportion of antibody-secreting plasma blasts and memory cells and an increasing amount of partially anergic, naive CD19 cells.

**Supplementary Information:**

The online version contains supplementary material available at 10.1186/s12979-023-00372-6.

## Background

Obesity is commonly characterized by excess accumulation of body fat and associated chronic low-grade inflammation. This inflammatory state is reflected in increased circulating levels of pro-inflammatory proteins. There are several connections between adipose tissue and the immune system, and it is postulated that chronic inflammation appears to influence the activation of immune cells [1, 2]. Special emphasis has been placed on the role of visceral adipose tissue (VAT) as an endocrine organ heavily influencing host metabolism [3, 4]. It places affected individuals at significant risk for a variety of severe diseases, including cardiovascular disease, type-2 diabetes mellitus, several cancer types, autoimmune conditions, respiratory diseases, and mental health issues [5–8].

Immunocompetence of both the innate and adaptive immune systems is key in eliminating pathogens and providing long-term immunity. A special role has been placed on B lymphocytes as key players in the humoral immune response and the formation of immunological memory [9, 10].

Alterations in B lymphocyte subsets have been described in a variety of conditions, such as autoimmune disorders, certain cancer types and primary immunodeficiencies but also in the context of immunosenescence [11, 12]. Similar to what has been observed in obese subjects, the reported shift in immune cell subsets places elderly individuals at increased risk for infection and impaired immune response [13].

Although there is considerable evidence of functional impairment of both the innate and adaptive immune systems in obesity, knowledge regarding the baseline immunological profile in obese individuals is still scarce. Existing studies have focused either predominantly on cells of the innate immune system or on cell-mediated immunity elicited by T cells. Less attention has been drawn to B lymphocytes, despite being crucial in specialized immune responses and the establishment of profound long-term immunity.

The aim of our study was to investigate the relationship between body fat percentage and B lymphocyte subpopulations in peripheral blood, taking a closer look at body fat distribution.

## Results

In total, 238 participants (69% females) were included in the study, and 94 participants were classified as obese based on their calculated body mass index (BMI ≥ 30 kg/m²). The median age of the participants was 48 (36; 56) years. In addition to expected differences in body composition measures, obese participants had higher blood pressure and were more frequently current or former smokers. Details of baseline characteristics are given in Table 1.

**Table 1:** Baseline characteristics of study participants

Characteristic	Total N = 238	Non-obese n = 144	Obese n = 94	p-value^1^
Sex: female (%)	164 (68.9)	108 (75.0)	56 (59.6)	0.015
Age in years	48.0 (36.0 ; 56.0)	48.0 (35.0 ; 54.3)	47.5 (36.8 ; 56.8)	0.523
BMI (kg/m²)	25.5 (22.7 ; 33.1)	23.2 (21.2 ; 24.9)	35.8 (32.4 ; 39.7)	< 0.001
Visceral adipose tissue (%)	2.0 (1.2 ; 3.5)	1.5 (0.8 ; 2.0)	3.6 (2.8 ; 5.4)	< 0.001
Total body fat (%)	34.0 (27.2 ; 41.2)	29.7 (23.9 ; 34.3)	43.7 (36.8 ; 48.3)	< 0.001
Waist-to-Height Ratio	0.5 (0.45 ; 0.63)	0.47 (0.43 ; 0.50)	0.65 (0.61 ; 0.7)	< 0.001
Systolic BP (mmHG)	116 (106.0; 127.5)	110 (102.5 ; 119.6)	126.5 (115.5 ; 135.5)	< 0.001
Diastolic BP (mmHG)	77 (70.5 ; 85.0)	73.8 (67.5 ; 78.5)	83 (78.0 ; 91.5)	< 0.001
Alcohol consumption^2^	0.3 (0.1 ; 0.7)	0.3 (0.1 ; 0.7)	0.3 (0.1 ; 0.7)	0.671
Autoimmune disease	46 (19.3)	27 (18.8)	19 (20.2)	0.867
Allergy	110 (46.4)	69 (47.9)	41 (44.1)	0.595
Type 2 DM	10 (4.3)	5 (3.5)	5 (5.4)	0.523
Cardiovascular disease	11 (4.6)	5 (3.5)	6 (6.4)	0.351
Hypothyroidism	46 (19.9)	28 (20.1)	18 (19.6)	1.000
Smoking status (%):				
Current smoker	34 (14.3)	13 (9.0)	21 (22.3)	< 0.001
Former smoker	85 (35.7)	44 (30.6)	41 (43.6)	
Never smoker	119 (50.0)	87 (60.4)	32 (34.0)	
Education (%):				
No professional education	4 (1.7)	2 (1.4)	2 (2.1)	0.005
Professional education	162 (68.1)	88 (61.1)	74 (78.7)	
Academic education	72 (30.3)	54 (37.5)	18 (19.1)	

Data are presented either as absolute numbers (percentages) or median (interquartile range: Q1; Q3)

^1^Test of difference between groups: two-sided Fisher’s exact test for categorical variables, Mann‒Whitney U test for continuous variables

^2^ Alcohol consumption: alcoholic beverages per day

Obese: BMI ≥ 30; non-obese: BMI < 30

Abbreviations: BMI: body mass index, BP: blood pressure, DM: diabetes mellitus

### Primary analysis

In the following sections, VAT and total body fat (TBF) represent the respective fat content relative to body weight (in %). Reported $\sigma$-standardized point estimates and 95% confidence intervals are given on the log_2_-scale, and presented p-values are false discovery rate (FDR) adjusted.

**Fig. 1:**
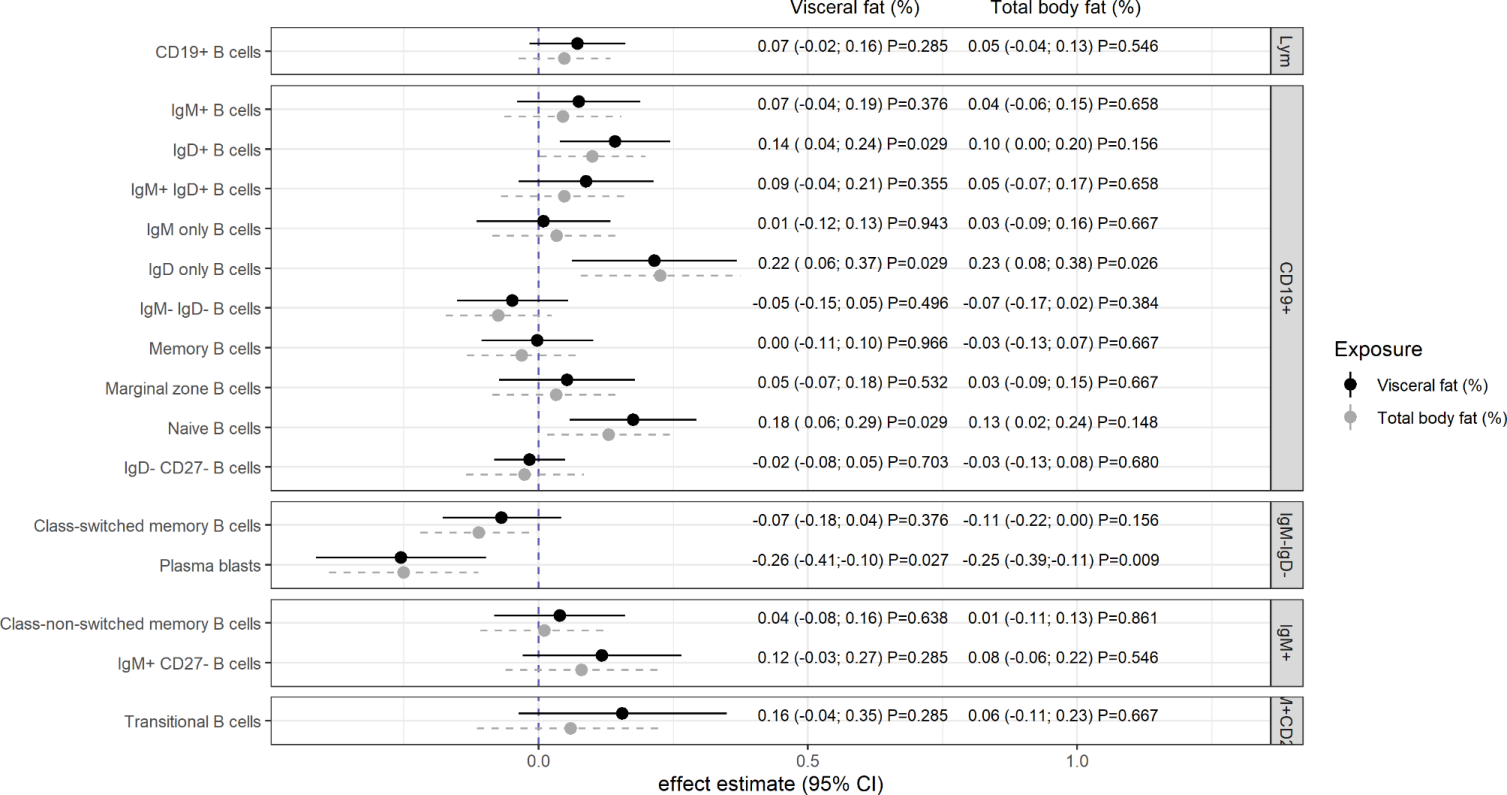
Association between visceral fat as well as total body fat and relative frequencies of B lymphocyte subsets in peripheral blood Relative frequencies (%) were calculated on total leukocyte counts. Effect estimates were derived from linear random intercept models adjusted for age, sex, smoking, alcohol consumption, education, study day and hypothyroidism. Estimates are presented on the log_2_-scale, exposure variables were $\sigma$-standardized; reported p-values are adjusted for multiple testing (FDR approach). Abbreviations: CI: confidence interval, Ig: immunoglobulin

VAT was significantly positively associated with the relative frequencies of IgD only B cells (β = 0.22; 95% CI: 0.06; 0.37; p = 0.029), IgD^**+**^ B cells (β = 0.14; 95% CI: 0.04; 0.24; p = 0.029) and naive B cells among total leukocytes (β = 0.18; 95% CI: 0.06; 0.29; p = 0.029) (Fig. 1). A strong negative association was found with plasma blast frequency (β = -0.26; 95% CI: -0.41; -0.10; p = 0.027).

TBF only yielded associations with IgD only B cells (β = 0.23; 95% CI: 0.08; 0.38; p = 0.026) and plasma blast frequencies (β = -0.25; 95% CI: -0.39; -0.11; p = 0.009) after adjusting for multiple testing. An association with IgD^**+**^ B cells was observed for VAT but not for TBF. Altogether, VAT showed slightly stronger point estimates in the majority of findings (Fig. 1). There were no further associations between VAT or TBF and the relative frequencies of B lymphocytes.

Within the sensitivity analysis, waist-to-height ratio (WHtR) confirmed the findings for VAT by showing similar but slightly stronger effect estimates (Supplementary Fig. 1). Additionally, WHtR was positively associated with the frequency of IgM^**+**^ CD27^**−**^ B cells. Analogously, the findings for the associations between TBF and immune cell frequencies could also be shown by using BMI as an exposure (Supplementary Fig. 2). Further positive associations with BMI were found for naive and IgD expressing B cells.

### Secondary analysis

**Fig. 2:**
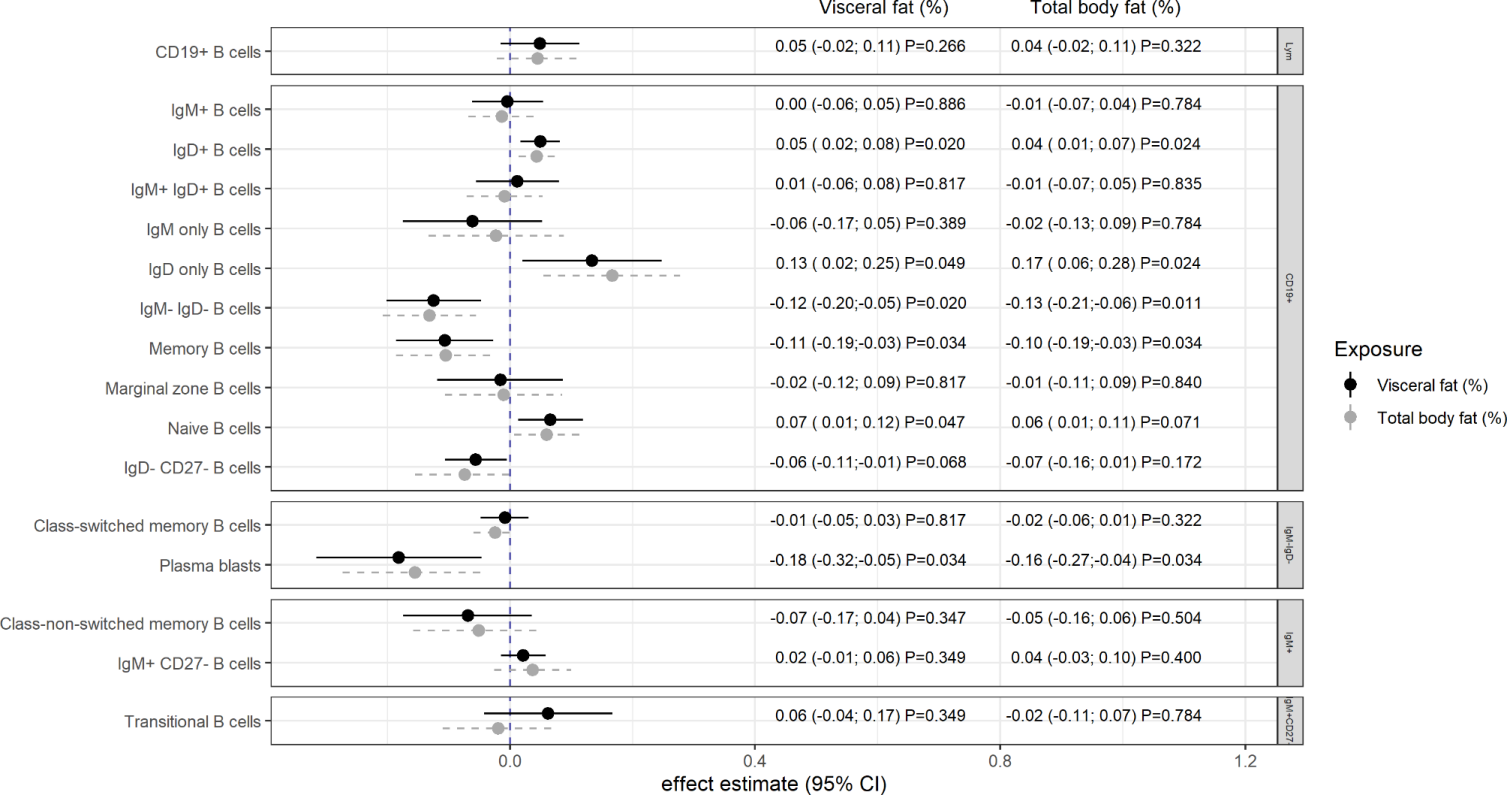
Association between visceral fat as well as total body fat and relative frequencies of B lymphocyte subsets (gated). Relative frequencies (%) were calculated on the number of cells identified within the corresponding gate (lymphocytes, CD19+, IgM+, IgM-IgD-, IgM + CD27-, respectively). Effect estimates were derived from linear random intercept models adjusted for age, sex, smoking, alcohol consumption, education, study day and hypothyroidism. Visceral and total body fat were used as percentages of total body weight and were $\sigma$-standardized. Estimates are presented on the log_2_-scale, and reported p-values are adjusted for multiple testing (FDR approach). Abbreviations: CI: confidence interval, Ig: immunoglobulin, Ly: lymphocytes

Analyses of immune cell frequencies relative to lymphocyte count of the respective gate showed that within the CD19^**+**^ gate, VAT and TBF were positively related to frequencies of both IgD only and IgD^**+**^ cells (Fig. 2). On the other hand, strong negative associations were observed between these body composition measures and memory B cells. VAT, but not TBF, was further associated with naive B cells in the gated analysis (Fig. 2).

Within the IgM^**−**^IgD^**−**^ gate, both VAT and TBF showed strong negative associations with plasma blast frequencies. Throughout the analyses, VAT and TBF presented very similar effect estimates. The only exception was found for the frequency of transitional B cells among IgM^**+**^CD27^**−**^ cells. In this case, we observed effect estimates in opposite directions for VAT and TBF, although none of them was significant at an $\alpha$-level of 0.05 (Fig. 2).

The WHtR-based results mainly supported the associations found for VAT (Supplementary Fig. 3). Again, the observed p-values were similar or stronger for WHtR than for VAT. While the adjusted β-estimate of WHtR on the relative frequency of IgD only B cells was not as strong as that observed for VAT, an additional positive association was found for CD19^**+**^ cells among the total lymphocyte count. The directions of the associations were inconsistent in the case of non-significant relationships with transitional B cells. The results for BMI were in line with those for TBF but with additional associations regarding naive B and total CD19^**+**^ cells (Supplementary Fig. 4).

## Discussion

In this analysis based on data from the MeGA study, we showed that both VAT and TBF are associated with the frequency of specific B cell subpopulations in peripheral blood. We found significant associations between body fat percentage and naive B lymphocytes (VAT), IgD-expressing B cells (VAT), the frequency of plasma blasts (VAT, TBF), and the relative frequencies of IgD only B cells (VAT, TBF) among total leukocyte counts.

Our findings are partially in line with previous research on this topic, but there are also some diverging results reported in prior studies. Wijngaarden et al., for example, compared the absolute and relative frequencies of immune cell subsets between morbidly obese people with and without metabolic syndrome (n = 117 and n = 127, respectively) and lean controls and found a significant increase in plasma blasts among morbidly obese subjects. Contrary to these results, our findings clearly suggest an inverse association between body fat, WHtR and BMI, and plasma blast frequency. Of note, Wijngaarden et al. observed this increase only in absolute cell counts, and the effect diminished when relative frequencies were considered [14]. Significant deviations from our findings could be due not only to the presence of metabolic syndrome in a substantial proportion of subjects but also to differences in the selection of participants. Whereas subjects with significant medical conditions were excluded from our study to draw inferences on the general, healthy population, Wijngaarden et al. did not apply exclusion criteria regarding comorbidities and medication use. Last, due to the purely cross-sectional nature of their study (no repeated measurements), findings may be more susceptible to obscure underlying infections or short-term challenges to the immune system that would amplify the generation of antibody-secreting plasma blasts and might explain discrepancies with our findings.

In contrast, our findings strongly support the observations made by Garner-Spitzer et al., who investigated B lymphocyte frequencies and antibody titers among obese subjects and lean controls in the context of tick-born encephalitis vaccine booster [15]. They determined B cell subsets prior to vaccination and one week, four weeks and six months after vaccination. They reported lower frequencies of memory B cells and plasma blasts but an expansion of naive B cells in obese participants prior to vaccination, which corresponds to our findings. The present study confirmed the inverse association between body fat accumulation and antibody-secreting plasma blasts and memory B cells. It should be noted that humoral memory relies on continuous secretion of antibodies, which is provided to a substantial extent by long-living plasma cells residing in the bone marrow [16, 17]. The concentration of plasma blasts in peripheral blood may therefore not sufficiently reflect the functional immune status of an individual.

Recently, Frasca et al. demonstrated that CD27- IgD- B cells were detected significantly more frequently in the plasma of obese subjects than in lean subjects [18]. This cell subset is highly proinflammatory, and it was demonstrated that it is characterized by a higher expression of immune activation markers and of the transcription factor T-bet. Interestingly, T-bet^+^ B cells and their antibodies are associated with autoimmunity [18]. These cells accumulate in the adipose tissue of obese humans and mice, and their frequency correlates with the increase in obesity [2]. Furthermore, they drive inflammation and exacerbate metabolic disorders during obesity [2]. In our study, no significant associations between body fat and CD27- IgD- B were found. The reasons for these different results are highly speculative. Further research on the origin and function of this cell subset is needed to better understand the role of these B cells in healthy individuals and certain diseases [19].

To our knowledge, the present study is the first to report a positive association between body fat and membrane-bound immunoglobulin D, particularly for B cells expressing IgD in the absence of IgM (IgD only B cells). The role of membrane-bound IgD is still discussed in the literature, and explicit functions have only recently begun to be uncovered [20]. Whether and if so, what role can be attributed to IgD only B cells in this context is not clear and requires further study.

Furthermore, we found an inverse association between body fat and IgM^**−**^IgD^**−**^ frequencies among CD19^**+**^ cells. Our results are partially in line with the findings from Frasca et al., who reported decreased frequencies of class-switched memory B cells among obese subjects [21]. Despite consistent point estimate direction, we could not observe an association between VAT or TBF and class-switched memory B cells in our sample. However, by including cells that are class-switched independent of CD27 and the expression level of CD38, based on estimations that up to 21% of IgG and IgA cells in peripheral blood lack expression of CD27 [17], the inverse association became evident in our analyses for all exposure variables. In the present sample, the association between body fat and relative frequencies of IgM^**−**^IgD^**−**^ B cells was much stronger for CD19^**+**^ cells than for total leukocytes. Changing the reference population thus could lead to a relativization of the observed association, as we also observed a trend toward higher frequencies of total CD19^**+**^ cells with increasing body fat.

### Strengths and Limitations

By determining body fat percentages using bioelectrical impedance analysis, we were able to obtain more accurate insights into body composition and actual body fat distribution than by using standard anthropometric measures alone. The application of mixed-effects models allowed us to incorporate up to four measurements per subject, thus averaging over observed immune cell frequencies, ultimately resulting in a well-powered longitudinal study. The comparatively large sample size, combined with a relatively low dropout rate, reduces selection bias.

One of the main limitations of our study is that an interpretation of the results is difficult without understanding one of the biological and/or immunological and/or pathophysiological function of those B cell subsets in obesity. Also, quantitative comparability of our findings with other studies must be taken with caution, since we determined antibody concentration by titration and did not use standardized preformulated antibody solutions as utilized in diagnostics, which might have influenced the number of detected cells and subsequently derived immune cell frequencies.

Furthermore, the data obtained in the present study are based on gating of the B cell population using the CD19 protein. This protein may also be expressed by other lymphocytes in circulation, such as natural killer cells (NK cells) [22]. NK cells have traditionally been classified as cells of the innate immune system. Recently, however, there is evidence of unexpected adaptive behavior and function of NK cells [23]. CD19, which belongs to the immunoglobulin superfamily, is generally considered a specific antigen of the B-cell lineage with high specificity, and it is not expected to be expressed by NK cells. However, previous studies have reported that normal NK cell subsets may occasionally show apparent expression of CD19 by flow cytometric measurements. The rare occurrence of this phenomenon raises the possibility that it may be misinterpreted as an abnormal population [24].Taking into account that CD19 + NK cells could represent a significant percentage of the lymphocyte population, potential masking of low levels of B lymphocytes or over-quantification of the B cell subset may occur by considering CD19 + NK cells as true B cells [22]. Therefore, when interpreting the results of this study, it should not be ignored that a significant source of CD19 + cells may actually be NK cells. Additionally, it cannot be excluded that the changes in the B cell pool may stem from other physiological alterations that may or may not be dependent on body fat mass, e.g., endocrine, nervous, or immune system alterations [25, 26]. Additionally, no isotype controls were available, and therefore, we could not distinguish between “low” and “negative” cells. Last, we have investigated the association between body fat and circulating B lymphocyte subsets, thus only providing an excerpt of the immunological profile of an individual.

## Conclusions

In conclusion, body fat, in particular VAT, appears to be associated with the relative abundance of B cell subpopulations in peripheral blood. Given the rising number of people living with excess body fat and obesity, it is key to fully understand the immunological profile of this population. Therefore, further research in this field is needed to better understand the interplay between immune cells and host metabolism and to investigate the molecular mechanisms of immune function in obesity.

## Methods

### Study design and population

Data from the observational study “Metabolic health and immune status in young obese subjects – Metabolische Gesundheit Augsburg” (MeGA) were used for the analyses. The single-center, population-based cohort study was prospectively conducted at the Chair of Epidemiology of the University of Augsburg in Germany and primarily aimed at investigating differences in immune status between obese subjects (BMI $\ge$ 30 kg/m²) and normal weight individuals (BMI preferably < 25 kg/m²). Participants (volunteers) were prospectively followed up for up to 12 months and underwent extensive examination and biosample collection at 3–4 study visits (baseline, 6 months, 9 months) during that period. A further study visit (28 days after vaccination) was thereby scheduled exclusively for subjects who received influenza vaccination by their treating physician. Recruitment took place between October 2018 and February 2021.

Participants were included in the study when they were at least 18 years old and gave written informed consent. Exclusion criteria comprised the use of corticosteroids or antibiotics three months prior to baseline examination, current use of immunosuppressive medicine, history of significant illness, and acute febrile illness. In total, 238 subjects were enrolled, of whom 202 completed the final follow-up, resulting in a dropout rate of 15.1% (Supplementary Fig. 5).

### Measures

Data collection included face-to-face interviews, anthropometric measurements, blood pressure measurements and blood sample collection in a fasting state (overnight fasting). Information on comorbidities, current use of medication, education, and health-related behaviors (e.g., smoking status, alcohol consumption) was obtained during interviews. Based on the anthropometric measurements comprising weight, height, waist- and hip circumference, we calculated BMI and WHtR. VAT and TBF were assessed via bioelectrical impedance analysis using a SECA mBCA (medical Body Composition Analyzer) 515 device. This device is designed for medical use, has been validated against Dual Energy X-Ray Absorptiometry, and its high accuracy in measuring fat content in different compartments has been proven in previous studies [27–29]. All examinations were carried out by trained study nurses in accordance with previously defined standard operating procedures.

### Flow cytometry

B lymphocyte subsets (including naive B cells, transitional B cells, marginal zone B cells, memory B cells, plasma blasts and membrane-bound immunoglobulins IgM, IgD) were determined in fresh EDTA-blood using fluorescence-based flow cytometry (Cytoflex LX flow cytometer, 6 lasers, Fa. Beckman Coulter). All cell types assessed, their input gates, and the frequency of their occurrence are listed in Table 2.

**Table 2:** Combinations and occurrence frequency of cell surface markers used to define B lymphocyte subpopulations

Cell type	Surface markers	Input gate	% in ungated cells	% in CD19 + B cells	% in the input gate
B cells	CD19+	Lym	3.28 (2.41; 4.44)	100 (100; 100)	10.96 (8.61; 13.88)
Naive B cells	CD19 + CD27- IgD+	CD19+	1.75 (1.20; 2.59)	56.80 (45.58; 66.36)	56.80 (45.58; 66.36)
Marginal zone B cells	CD19 + CD27 + IgD+	CD19+	0.45 (0.30; 0.68)	14.03 (9.43; 20.19)	14.03 (9.43; 20.19)
IgM + B cells	CD19 + IgM+	CD19+	1.73 (1.14; 2.50)	53.87 (42.57; 64.63)	53.87 (42.57; 64.63)
IgD + B cells	CD19 + IgD+	CD19+	2.32 (1.66; 3.24)	73.44 (64.73; 79.36)	73.44 (64.73; 79.36)
IgM + IgD + B cells	CD19 + IgM + IgD+	CD19+	1.49 (0.95; 2.19)	45.99 (34.44; 57.89)	45.99 (34.44; 57.89)
IgM only B cells	CD19 + IgM + IgD-	CD19+	0.20 (0.12; 0.30)	5.92 (4.00; 8.70)	5.92 (4.00; 8.70)
IgD only B cells	CD19 + IgM- IgD+	CD19+	0.73 (0.46; 1.17)	22.70 (15.11; 32.67)	22.70 (15.11; 32.67)
IgM- IgD- B cells	CD19 + IgM- IgD-	CD19+	0.66 (0.46; 0.96)	20.48 (15.22; 26.79)	20.48 (15.22; 26.79)
Class-switched memory B cells	CD19 + IgM- IgD- CD27 + CD38^low^	IgM- IgD-	0.39 (0.27; 0.58)	11.88 (8.53; 16.78)	60.12 (52.50; 67.51)
Plasma blasts	CD19 + IgM- IgD- CD27 + CD38^high^	IgM- IgD-	0.02 (0.01; 0.03)	0.54 (0.31; 0.97)	2.70 (1.60; 4.36)
Class non-switched memory B cells	CD19 + IgM + CD27 + CD38-	IgM+	0.45 (0.31; 0.69)	14.19 (9.68; 20.18)	28.50 (18.22; 41.79)
Transitional B cells	CD19 + IgM + CD27- CD24 + CD38+	IgM + CD27-	0.13 (0.07; 0.24)	3.99 (2.28; 6.61)	12.20 (8.20; 16.77)
Memory B cells	CD19 + CD27 + CD38-	CD19+	0.97 (0.67; 1.40)	30.38 (22.05; 40.64)	30.38 (22.05; 40.64)
IgD-CD27- B cells	CD19 + CD27- IgD-	CD19+	0.35 (0.21; 0.50)	9.37 (6.84; 12.45)	9.37 (6.84; 12.45)
IgM + CD27- B cells	CD19 + IgM + CD27-	IgM+	1.23 (0.73; 2.00)	35.96 (23.57; 47.90)	69.26 (54.95; 79.60)

The frequency of occurrence is presented as the median and interquartile range

Note: IgD + B cells consisted of IgM + IgD + and IgM- IgD + B cells (analogous for IgM + B cells)

**Fig. 3:**
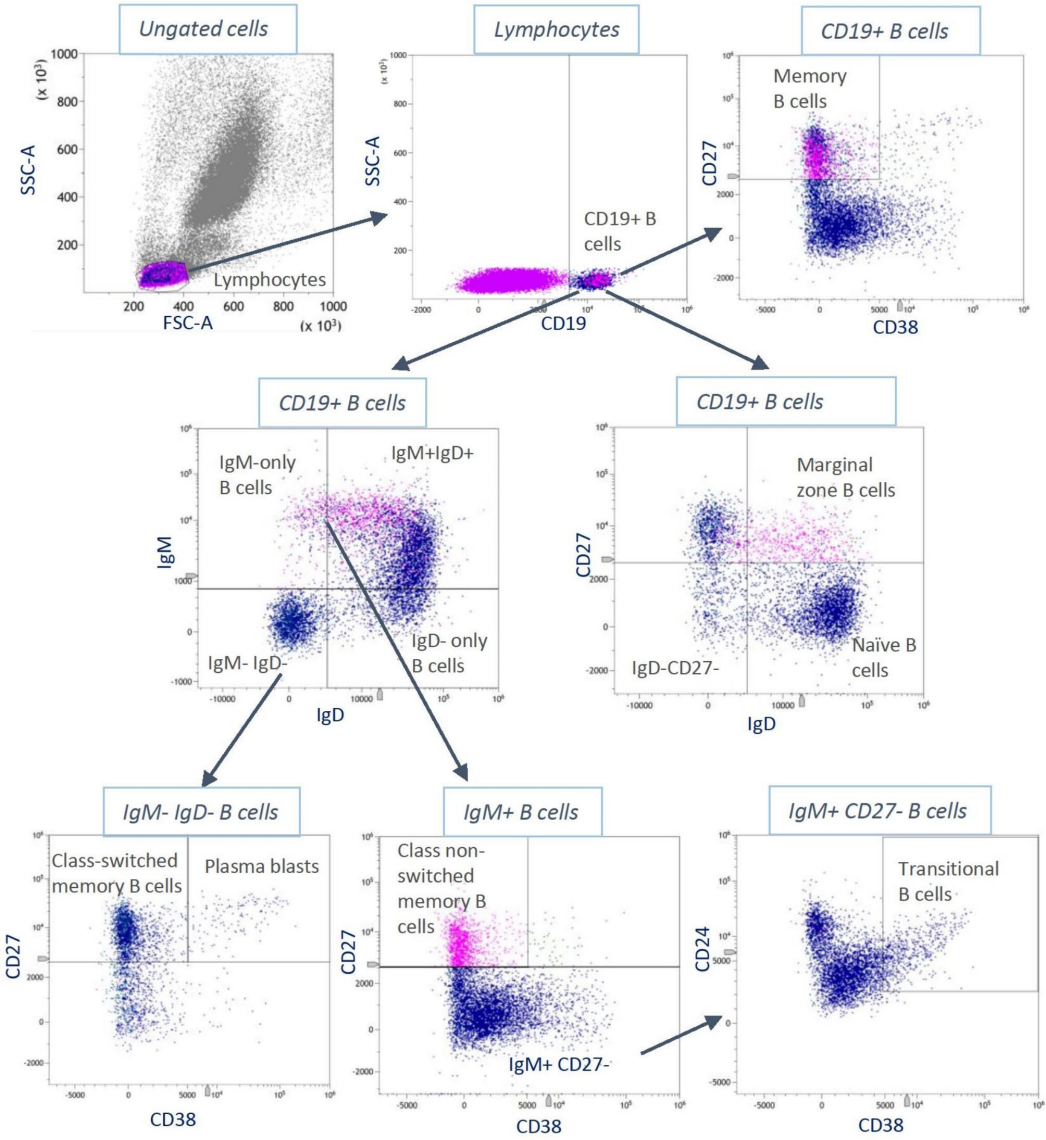
Applied gating strategy. Gating strategy used for the identification of B lymphocyte subsets (flow cytometry) according to Streitz et al. All B cells expressing IgM are colored blue and all B cells expressing IgD are colored red

After lysis of erythrocytes with VersaLyse Lysing Solution from Fa. Beckman Coulter, leukocytes were isolated in several washing steps. Prior to antibody staining, leukocytes were treated with an FC receptor block (Fa. Miltenyi Biotec) to avoid non-specific antibody binding. Staining was performed with fluorescence-labeled liquid anti-human antibodies (anti-CD19 PerCP-Cy5.5, anti-CD21 PE, anti-CD24 BV605, CD27 PE-Cy7, anti-CD38 APC, anti-IgM Bv421, anti-IgD BB515 (all Fa. BD Bioscience). The correct antibody concentration was predetermined by titration. The antibody-coupled cells were fixed using IO-Test 3 Fixative Solution from Beckman Coulter before being analyzed by flow cytometry. Light scattering measurements were used to determine the size and granularity of the cells, and fluorescence labeling allowed further differentiation and quantification of immune cell fractions based on the expression of cell surface antigens. Single color staining was included for compensation purposes, and an appropriate number of cells (at least 100,000 lymphocytes) was recorded per staining. The applied gating strategy for the identification of subpopulations is presented in Fig. 3. Kaluza software (Fa. Beckman Coulter) was used for subsequent data analysis. For the present study, the applied gating strategy was adopted from a publication from Streitz et al. (ONE study), which was available at the beginning of the MeGA study [30]. According to that study, leukocytes were classified based on CD45 expression versus side scatter. In all panels, absolute numbers of subpopulations were calculated using the CD45 + leukocyte “backbone” in combination with the whole blood count of all samples [30]. For identification of the B-cell population, mainly the CD19 protein was used. The flow cytometry measurements were highly standardized (one laboratory, same procedure from beginning of the study until the end, only two different persons carrying out the measurements who were permanently in close contact).

### Statistical analyses

Categorical and continuous baseline characteristics were reported as percentages and medians with interquartile ranges, respectively. Differences in characteristics between non-obese (BMI $<$ 30 kg/m²) and obese subjects (BMI $\ge$ 30 kg/m²) were assessed using two-sided Fisher’s exact test for categorical variables and two-sided Mann‒Whitney U test for continuous variables at a significance level of 0.05.

Linear mixed models with random intercepts based on maximum likelihood estimation were used to investigate the association between body fat percentage and immune cell frequencies, accounting for individual-specific changes over time. As primary outcomes, we calculated relative frequencies of B lymphocyte subsets among total leukocyte counts in percent. For secondary analysis, percentages were calculated on the corresponding gate. The total number of cells identified within the gate was thereby considered as the reference, corresponding to 100%. Regarding the skewness of distributions, all outcome variables were log_2_-transformed, and outliers with more than three standard deviation differences from the mean were excluded from the respective analysis.

We used VAT and TBF as primary exposure variables. Both measures were calculated as percentages of total body weight to ensure comparability across subjects independently of body weight. In sensitivity analyses, we used WHtR and BMI as surrogates of body fat measures to validate the results and increase the comparability of findings with other studies. Therefore, we compared findings in VAT with WHtR and findings in TBF with BMI in the sensitivity analyses.

Confounders included in the analyses were determined using direct acyclic graphs based on the literature and disjunctive cause criterion (Supplementary Fig. 6) [31]. Thus, data on age, sex, education (no professional education, professional education, academic education), smoking status (current smoker, former smoker, never smoker), alcohol consumption and hypothyroidism (presence: yes or no) were used for the analyses to adjust for potential confounding.

Continuous variables were used as such, and body fat percentages, WHtR and BMI were $\sigma$-standardized to enable direct comparison of effect estimates. The linearity assumption was tested using restricted cubic splines for continuous covariables with a covariable-specific number of knots determined in compliance with the Akaike Information Criterion (AIC). The resulting models were tested against the random intercept model comprising only linear terms using the likelihood ratio test. Based on the AIC, linearity could be assumed for all continuous predictors. Homoscedasticity and normal distribution of residuals were assessed graphically using residual scatter plots (standardized residuals vs. fitted values) and Q‒Q-Plots, respectively. Continuous covariables were tested for multicollinearity by calculating the correlation matrix as well as the variance inflation factor.

Models were tested for significant interactions between the primary exposure variables and each covariable using the Wald test for the respective interaction terms. Since loss to follow-up was considered low and no pattern in missing observations was detected, we considered the missing value mechanism to be missing completely at random and subsequently performed regression analyses using all available information, including that of drop-outs.

Based on a type I error probability of 0.05, presented p-values were adjusted for multiple testing by controlling the false discovery rate (FDR approach). All derived effect estimates (β, 95% Confidence Interval) can be interpreted as the expected change in the log_2_-transformed outcome associated with one standard deviation increase in the primary exposure variable. Statistical analyses were performed using R version 4.1.3 (packages (version): lme4 (1.1–29), Matrix (1.4-0), rms (6.3-0), tidyverse (1.3.1), MuMIn (1.46.0), lmerTest (3.1-3), AER (1.2-9), ggplot2 (3.3.5), performance (0.9.1), dagitty (0.3-1)).

### Electronic supplementary material

Below is the link to the electronic supplementary material.

Supplementary Fig. 1: Association between visceral fat, respectively waist-to-height ratio, and relative frequencies of B lymphocyte subsets (ungated). Supplementary Fig. 2: Association between total body fat, respectively body mass index, and relative frequencies of B lymphocyte subsets (ungated). Supplementary Fig. 3: Association between visceral fat, respectively waist-to-height ratio, and relative frequencies of B lymphocyte subsets (gated). Supplementary Fig. 4: Association between total body fat, respectively body mass index, and relative frequencies of B lymphocyte subsets (gated). Supplementary Fig. 5: Participant flowchart. Supplementary Fig. 6: Simplified presentation of the directed acyclic graphs depicting the relationship between obesity and circulating immune cells.

## Data Availability

Data are not publicly available but can be applied for through an individual project agreement with the Chair of Epidemiology, Medical Faculty at University of Augsburg. The datasets used and/or analyzed during the current study are available from the corresponding author on reasonable request.

## Declarations

The study (“Metabolische Gesundheit Augsburg Studie”) was registered at the DRKS (Deutsches Register Klinische Studien) under the number DRKS00015784 (URL: https://drks.de/search/de).

## Abbreviations

AIC: Akaike Information Criterion
BMI: Body mass index
CI: Confidence interval
FDR: False discovery rate
mBCA: Medical Body Composition Analyzer
NK: Natural killer
TBF: Total body fat
VAT: Visceral adipose tissue
WHtR: Waist-to-height ratio

## References

[1] de Heredia FP, Gomez-Martinez S, Marcos A (2012). Obesity, inflammation and the immune system. Proc Nutr Soc.

[2] Hagglof T, Vanz C, Kumagai A, Dudley E, Ortega V, Siller M (2022). T-bet(+) B cells accumulate in adipose tissue and exacerbate metabolic disorder during obesity. Cell Metab.

[3] Ellulu MS, Patimah I, Khaza’ai H, Rahmat A, Abed Y (2017). Obesity and inflammation: the linking mechanism and the complications. Arch Med Sci.

[4] Grant RW, Dixit VD (2015). Adipose tissue as an immunological organ. Obes (Silver Spring).

[5] Organization WH. New WHO report: Europe can reverse its obesity “epidemic”2022 03.05.2022; (23.05.2022). Available from: https://www.euro.who.int/en/media-centre/sections/press-releases/2022/new-who-report-europe-can-reverse-its-obesity-epidemic.

[6] Andrade FB, Gualberto A, Rezende C, Percegoni N, Gameiro J, Hottz ED. The weight of obesity in immunity from Influenza to COVID-19. Front Cell Infect Microbiol. 2021;11. 10.3389/fcimb.2021.638852 PMC8011498 33816341

[7] Fox CS, Massaro JM, Hoffmann U, Pou KM, Maurovich-Horvat P, Liu CY (2007). Abdominal visceral and subcutaneous adipose tissue compartments: association with metabolic risk factors in the Framingham Heart Study. Circulation.

[8] Ritchie SA, Connell JM (2007). The link between abdominal obesity, metabolic syndrome and cardiovascular disease. Nutr Metab Cardiovasc Dis.

[9] Abbas AK, Lichtman AH, Pillai S. Cellular and Molecular Immunology. 10th ed. Jeremy Bowes; 2022. pp. 6–11.

[10] Abbas AK, Lichtman AH, Pillai S. Cellular and Molecular Immunology. 10th ed. Jeremy Bowes; 2022. pp. 271–82.

[11] Aiello A, Farzaneh F, Candore G, Caruso C, Davinelli S, Gambino CM et al. Immunosenescence and its Hallmarks: how to oppose aging strategically? A review of potential options for therapeutic intervention. Front Immunol. 2019;10. 10.3389/fimmu.2019.02247 PMC6773825 31608061

[12] Crooke SN, Ovsyannikova IG, Poland GA, Kennedy RB, Immunosenescence (2019). A systems-level overview of immune cell biology and strategies for improving vaccine responses. Exp Gerontol.

[13] Frasca D, Diaz A, Romero M, Blomberg BB (2017). Ageing and obesity similarly impair antibody responses. Clin Exp Immunol.

[14] Wijngaarden LH, van der Harst E, Klaassen RA, Dunkelgrun M, Kuijper TM, Klepper M (2021). Effects of morbid obesity and metabolic syndrome on the composition of circulating Immune subsets. Front Immunol.

[15] Garner-Spitzer E, Poellabauer EM, Wagner A, Guzek A, Zwazl I, Seidl-Friedrich C (2020). Obesity and sex affect the Immune responses to Tick-Borne Encephalitis Booster Vaccination. Front Immunol.

[16] Radbruch A, Muehlinghaus G, Luger EO, Inamine A, Smith KG, Dörner T (2006). Competence and competition: the challenge of becoming a long-lived plasma cell. Nat Rev Immunol.

[17] Perez-Andres M, Paiva B, Nieto WG, Caraux A, Schmitz A, Almeida J (2010). Human peripheral blood B-cell compartments: a crossroad in B-cell traffic. Cytometry B Clin Cytom.

[18] Frasca D, Diaz A, Romero M, Blomberg BB (2021). Phenotypic and functional characterization of double negative B cells in the blood of individuals with obesity. Front Immunol.

[19] Beckers L, Somers V, Fraussen J (2023). IgD(-)CD27(-) double negative (DN) B cells: Origins and functions in health and disease. Immunol Lett.

[20] Gutzeit C, Chen K, Cerutti A (2018). The enigmatic function of IgD: some answers at last. Eur J Immunol.

[21] Frasca D, Ferracci F, Diaz A, Romero M, Lechner S, Blomberg BB (2016). Obesity decreases B cell responses in young and elderly individuals. Obes (Silver Spring).

[22] Korol C, Rossi J, Sanz M, Bernasconi A (2015). NK cells expressing the B cell antigen CD19: expanding the phenotypical characterization and the potential consequences from misinterpretation of this subset population. Cytometry B Clin Cytom.

[23] Sun JC, Beilke JN, Lanier LL (2009). Adaptive immune features of natural killer cells. Nature.

[24] Soma L, Wu D, Chen X, Edlefsen K, Fromm JR, Wood B (2015). Apparent CD19 expression by natural killer cells: a potential confounder for minimal residual disease detection by flow cytometry in B lymphoblastic leukemia. Cytometry B Clin Cytom.

[25] You Z, Liu B, Qi H (2022). Neuronal regulation of B-cell immunity: anticipatory immune posturing?. Neuron.

[26] Eibel H, Kraus H, Sic H, Kienzler AK, Rizzi M (2014). B cell biology: an overview. Curr Allergy Asthma Rep.

[27] Bosy-Westphal A, Schautz B, Later W, Kehayias JJ, Gallagher D, Muller MJ (2013). What makes a BIA equation unique? Validity of eight-electrode multifrequency BIA to estimate body composition in a healthy adult population. Eur J Clin Nutr.

[28] Day K, Kwok A, Evans A, Mata F, Verdejo-Garcia A, Hart K et al. Comparison of a Bioelectrical Impedance device against the reference Method Dual Energy X-Ray Absorptiometry and Anthropometry for the evaluation of body composition in adults. Nutrients. 2018;10(10). 10.3390/nu10101469 PMC6213252 30308974

[29] Lahav Y, Goldstein N, Gepner Y (2021). Comparison of body composition assessment across body mass index categories by two multifrequency bioelectrical impedance analysis devices and dual-energy X-ray absorptiometry in clinical settings. Eur J Clin Nutr.

[30] Streitz M, Miloud T, Kapinsky M, Reed MR, Magari R, Geissler EK (2013). Standardization of whole blood immune phenotype monitoring for clinical trials: panels and methods from the ONE study. Transpl Res.

[31] VanderWeele T, Robins J (2010). Signed directed acyclic graphs for causal inference. J Royal Stat Soc Ser B.

