# Supplementary material for: Association between body fat distribution and B-lymphocyte subsets in peripheral blood
Source: Immun Ageing. 2023 Sep 13;20:47. doi: 10.1186/s12979-023-00372-6 (PMC10498588; doi:10.1186/s12979-023-00372-6)
Supplement: Supplementary file 1 — Supplementary Fig. 1: Association between visceral fat, respectively waist-to-height ratio, and relative frequencies of B lymphocyte subsets (ungated). Supplementary Fig. 2: Association between total body fat, respectively body mass index, and relative frequencies of B lymphocyte subsets (ungated). Supplementary Fig. 3: Association between visceral fat, respectively waist-to-height ratio, and relative frequencies of B lymphocyte subsets (gated). Supplementary Fig. 4: Association between total body fat, respectively body mass index, and relative frequencies of B lymphocyte subsets (gated). Supplementary Fig. 5: Participant flowchart. Supplementary Fig. 6: Simplified presentation of the directed acyclic graphs depicting the relationship between obesity and circulating immune cells. [file 12979_2023_372_MOESM1_ESM.docx]

Supplementary Material

**Association between body fat distribution and B-lymphocyte subsets in peripheral blood**

Pia Prechtl, Timo Schmitz, Nicole Pochert, Claudia Traidl-Hoffmann, Jakob Linseisen, Christa Meisinger, Dennis Freuer*

*** Corresponding author:** Dennis Freuer: [dennis.freuer@med.uni-augsburg.de](mailto:dennis.freuer@med.uni-augsburg.de)

| Supplementary Figure 1 | Association between visceral fat, respectively waist-to-height ratio, and relative frequencies of B lymphocyte subsets (ungated) |
| --- | --- |
| Supplementary Figure 2 | Association between total body fat, respectively body mass index, and relative frequencies of B lymphocyte subsets (ungated) |
| Supplementary Figure 3 | Association between visceral fat, respectively waist-to-height ratio, and relative frequencies of B lymphocyte subsets (gated) |
| Supplementary Figure 4 | Association between total body fat, respectively body mass index, and relative frequencies of B lymphocyte subsets (gated) |
| Supplementary Figure 5 | Participant flowchart |
| Supplementary Figure 6 | Simplified presentation of the directed acyclic graphs depicting the relationship between obesity and circulating immune cells |


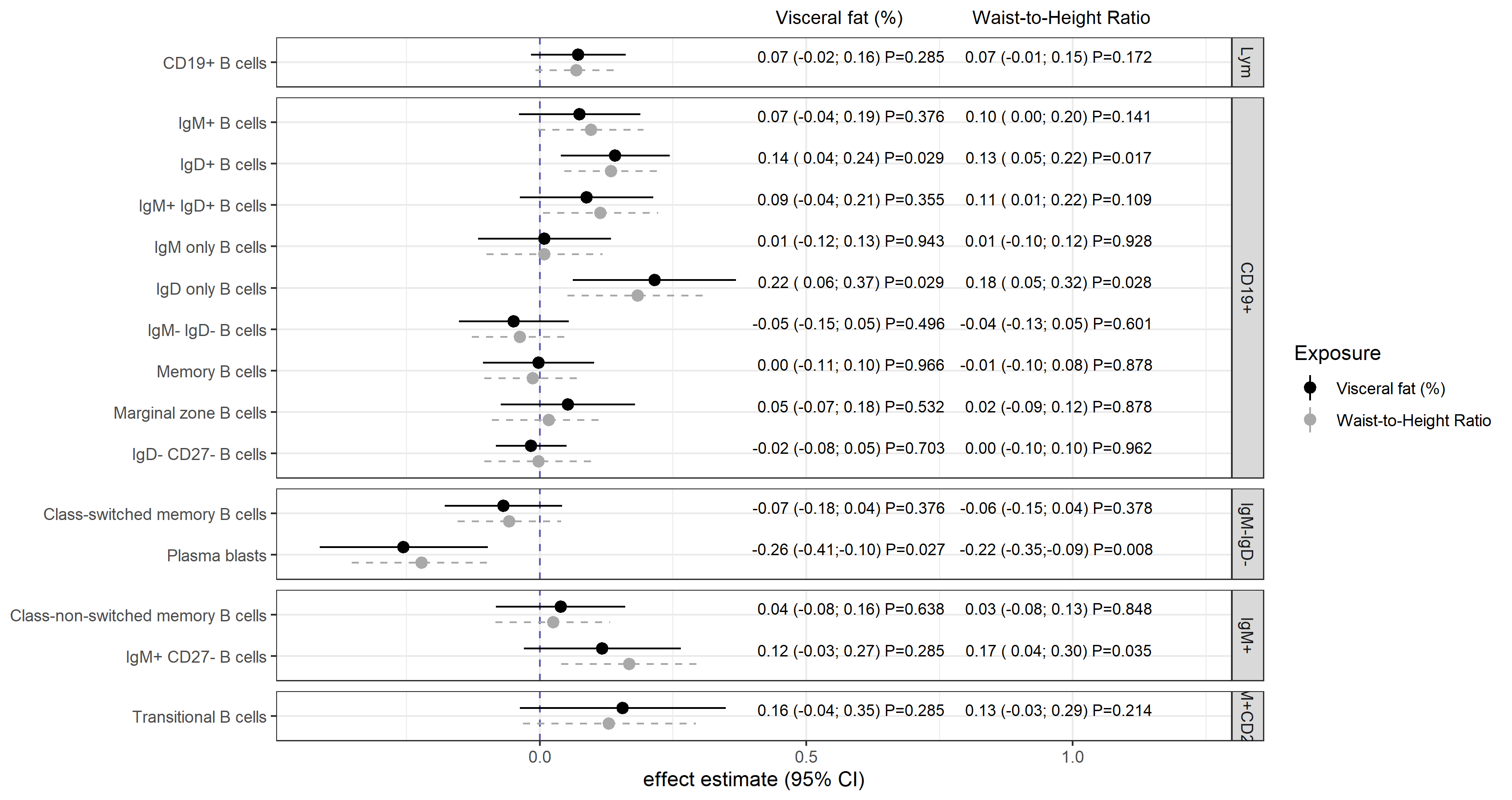


Supplementary Figure 1 Association between visceral fat, respectively waist-to-height ratio, and relative frequencies of B lymphocyte subsets in peripheral blood

Relative frequencies (%) were calculated on total leukocyte counts.

Effect estimates were derived from linear random intercept models adjusted for age, sex, smoking, alcohol consumption, education, study day and hypothyroidism.

Estimates are presented on the log_2_-scale, exposure variables were $\sigma$-standardized; reported p-values are adjusted for multiple testing (FDR approach).

Abbreviations: CI: Confidence Interval, Ig: Immunoglobulin


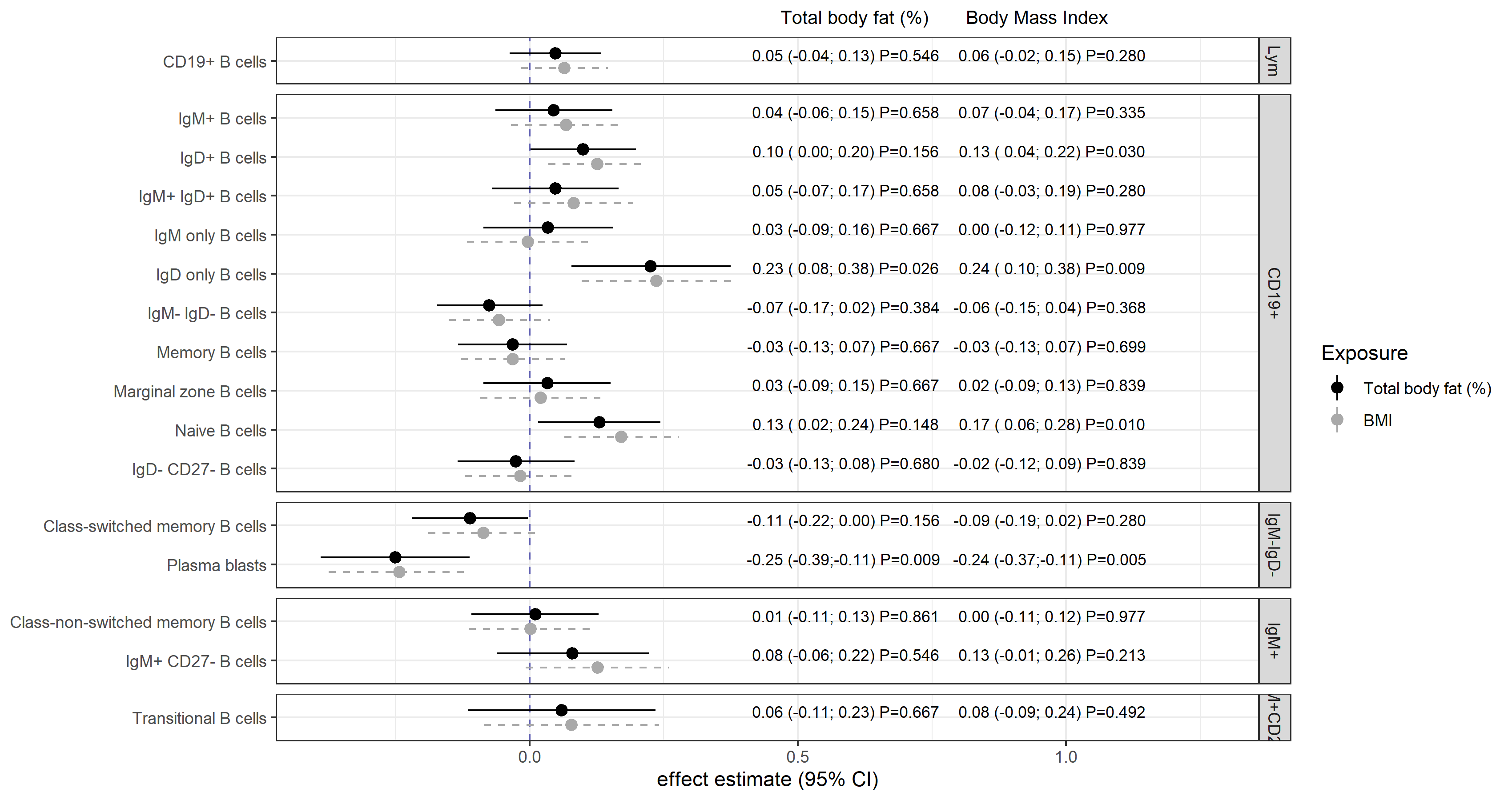


Supplementary Figure 2 Association between total body fat, respectively body mass index, and relative frequencies of B lymphocyte subsets in peripheral blood

Relative frequencies (%) were calculated on total leukocyte counts.

Effect estimates were derived from linear random intercept models adjusted for age, sex, smoking, alcohol consumption, education, study day and hypothyroidism.

Estimates are presented on the log_2_-scale, exposure variables were $\sigma$-standardized; reported p-values are adjusted for multiple testing (FDR approach).

Abbreviations: BMI: body mass index, CI: confidence interval, Ig: immunoglobulin


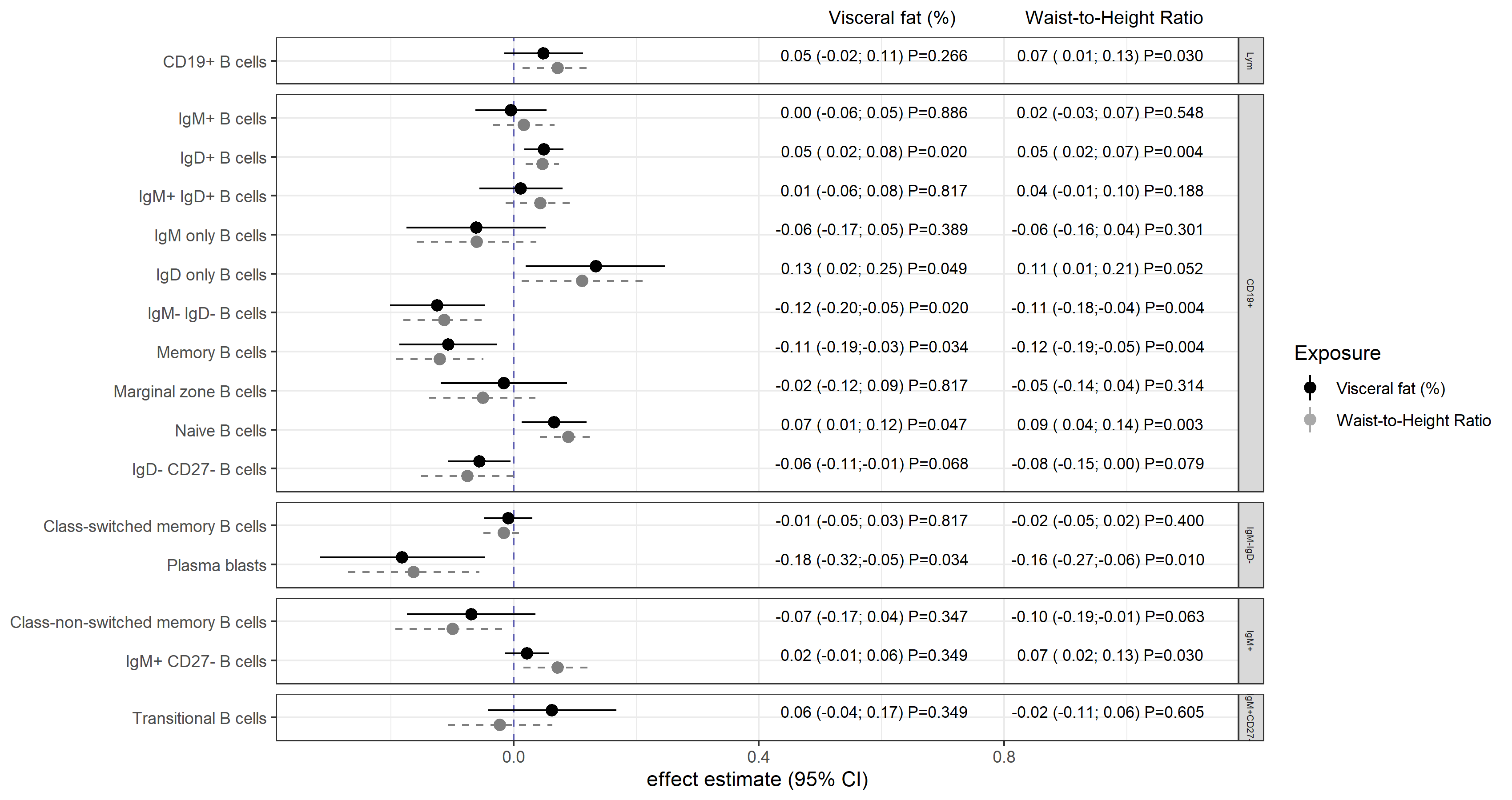


Supplementary Figure 3 Association between visceral fat, respectively waist-to-height ratio, and relative frequencies of B lymphocyte subsets (gated)

Relative frequencies (%) were calculated on the number of cells identified within the corresponding gate (CD19, IgM, IgM-IgD-, IgM+CD27-, Ly, respectively).

Effect estimates were derived from linear random intercept models adjusted for age, sex, smoking, alcohol consumption, education, study day and hypothyroidism.

Estimates are presented on the log_2_-scale, exposure variables were $\sigma$-standardized; reported p-values are adjusted for multiple testing (FDR approach).

Abbreviations: CI: confidence interval, Ig: immunoglobulin, Ly: lymphocytes


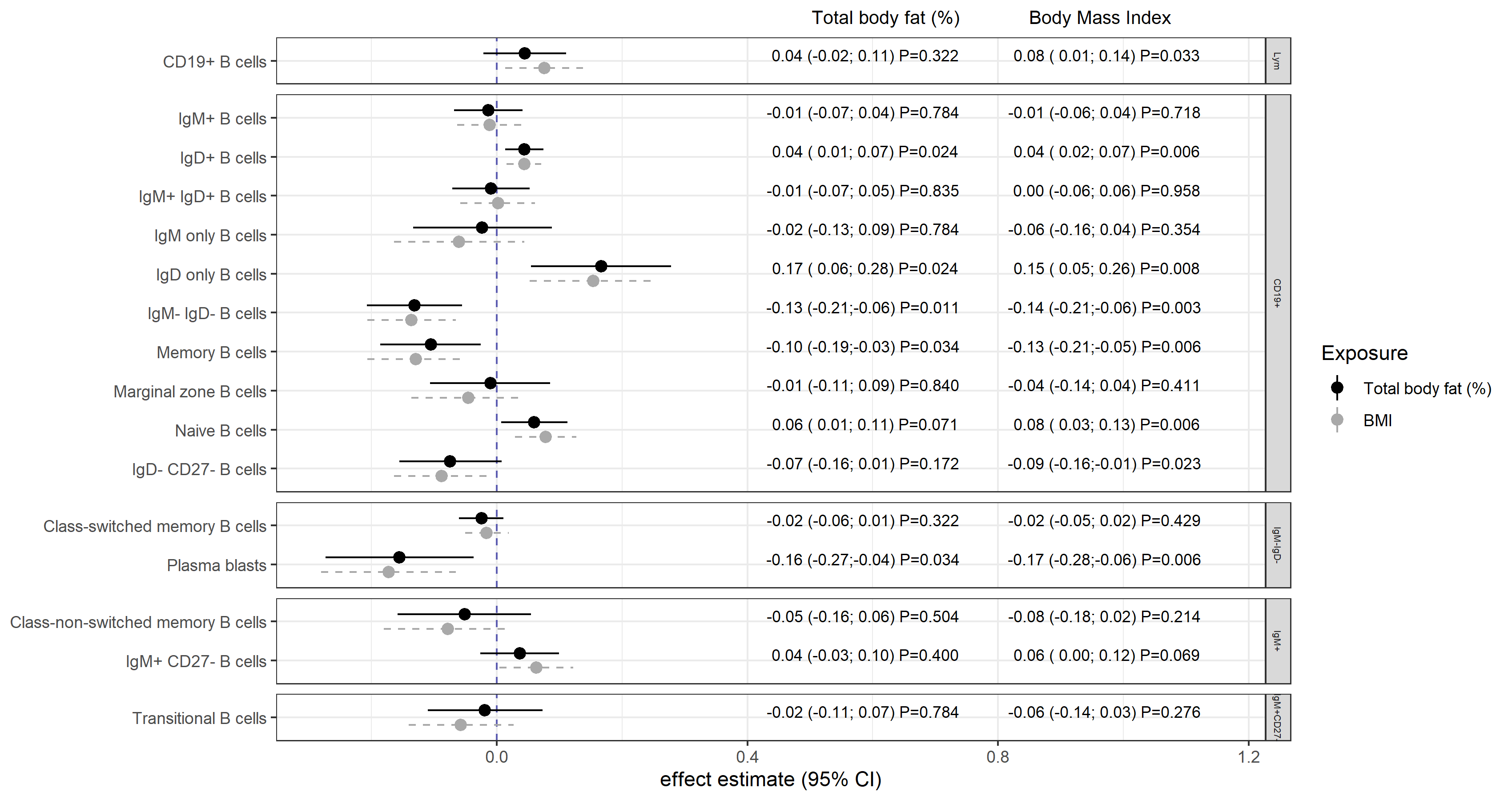


Supplementary Figure 4 Association between total body fat, respectively body mass index, and relative frequencies of B lymphocyte subsets (gated)

Relative frequencies (%) were calculated on the number of cells identified within the corresponding gate (CD19, IgM, IgM-IgD-, IgM+CD27-, Ly, respectively).

Effect estimates were derived from linear random intercept models adjusted for age, sex, smoking, alcohol consumption, education, study day and hypothyroidism.

Estimates are presented on the log_2_-scale, exposure variables were $\sigma$-standardized; reported p-values are adjusted for multiple testing (FDR approach).

Abbreviations: BMI: body mass index, CI: confidence interval, Ig: immunoglobulin, Ly: lymphocytes

**
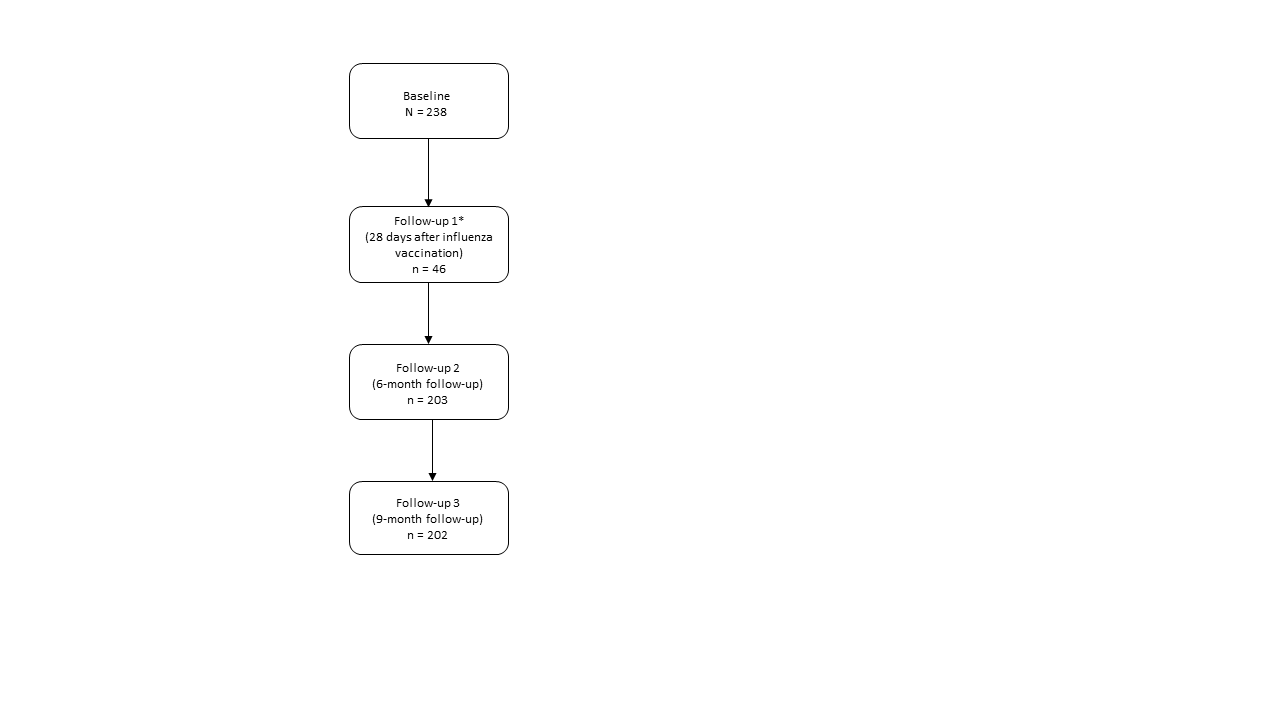
**

**Supplementary Figure 5** Participant flowchart

*Follow-up 1 was scheduled exclusively for participants receiving influenza vaccination


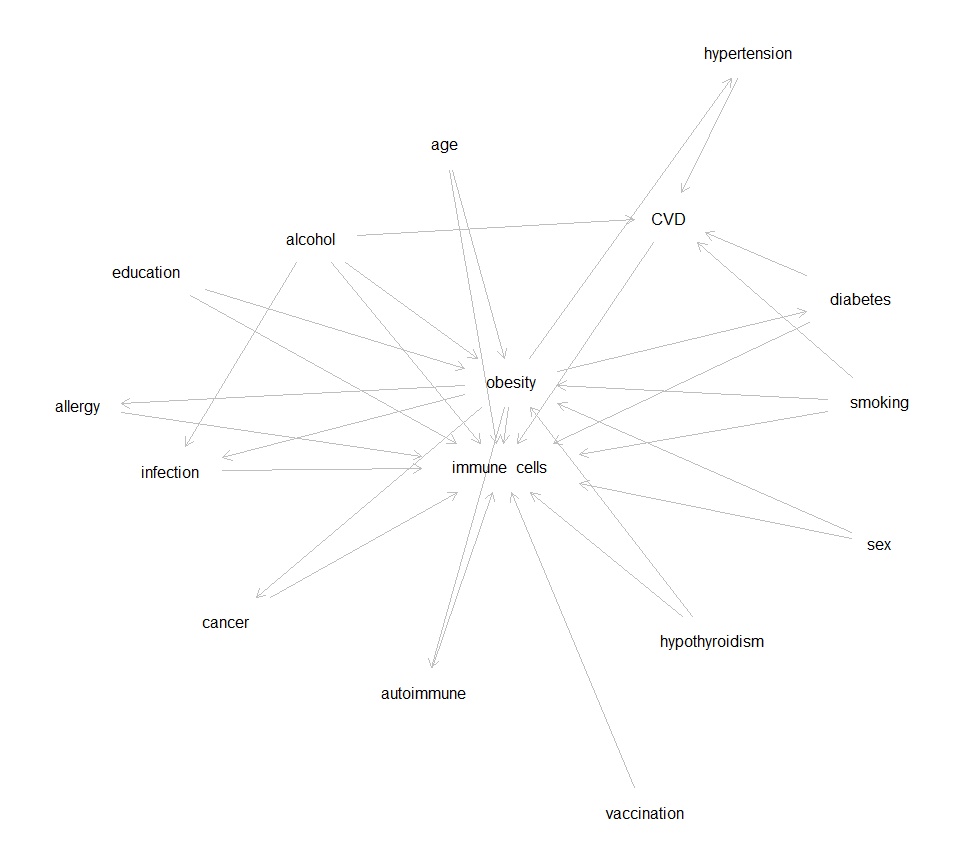


Supplementary Figure 6 Simplified presentation of the directed acyclic graphs depicting the relationship between obesity and circulating immune cells

Exposure: obesity, outcome: immune cell frequencies

Abbreviations: autoimmune: autoimmune conditions, CVD: cardiovascular disease
